# Association between striatal dopamine D_2_/D_3_ receptors and brain activation during visual attention: effects of sleep deprivation

**DOI:** 10.1038/tp.2016.93

**Published:** 2016-05-31

**Authors:** D Tomasi, G-J Wang, N D Volkow

**Affiliations:** 1 National Institute on Alcohol Abuse and Alcoholism, Bethesda, MD, USA; 2 National Institute on Drug Abuse, Bethesda, MD, USA

## Abstract

Sleep deprivation (SD) disrupts dopamine (DA) signaling and impairs attention. However, the interpretation of these concomitant effects requires a better understanding of dopamine’s role in attention processing. Here we test the hypotheses that D_2_/D_3_ receptors (D_2_/D_3_R) in dorsal and ventral striatum would distinctly regulate the activation of attention regions and that, by decreasing D_2_/D_3_, SD would disrupt these associations. We measured striatal D_2_/D_3_R using positron emission tomography with [^11^C]raclopride and brain activation to a visual attention (VA) task using 4-Tesla functional magnetic resonance imaging. Fourteen healthy men were studied during rested wakefulness and also during SD. Increased D_2_/D_3_R in striatum (caudate, putamen and ventral striatum) were linearly associated with higher thalamic activation. Subjects with higher D_2_/D_3_R in caudate relative to ventral striatum had higher activation in superior parietal cortex and ventral precuneus, and those with higher D_2_/D_3_R in putamen relative to ventral striatum had higher activation in anterior cingulate. SD impaired the association between striatal D_2_/D_3_R and VA-induced thalamic activation, which is essential for alertness. Findings suggest a robust DAergic modulation of cortical activation during the VA task, such that D_2_/D_3_R in dorsal striatum counterbalanced the stimulatory influence of D_2_/D_3_R in ventral striatum, which was not significantly disrupted by SD. In contrast, SD disrupted thalamic activation, which did not show counterbalanced DAergic modulation but a positive association with D_2_/D_3_R in both dorsal and ventral striatum. The counterbalanced dorsal versus ventral striatal DAergic modulation of VA activation mirrors similar findings during sensorimotor processing (Tomasi *et al.*, 2015) suggesting a bidirectional influence in signaling between the dorsal caudate and putamen and the ventral striatum.

## Introduction

Attention allows us to focus on one aspect of information (that is, the moving ball) while ignoring irrelevant information (that is, other moving objects in the scene), an ability severely compromised by sleep deprivation (SD).^1^ Attention engages a distributed network of brain regions for focusing on specific stimuli or the surroundings, and for resolving conflict between multiple cues.^2^ Several neurotransmitters are implicated in the modulation of these attention components, including cholinergic, noradrenergic and dopaminergic systems.^3, 4^ During the last decade, there has been an increased interest on the role of dopamine (DA) in the modulation of attention^5^ as stimulant medications enhance DA signaling in the human brain^6, 7, 8^ and improve attention under excessive sleepiness.^9, 10^

Previous studies have shown that SD decreases striatal D_2_/D_3_R availability, impairs performance and alters brain activation during attention tasks.^11, 12, 13, 14, 15, 16, 17^ Specifically, SD has been shown to impair performance to attention demanding cognitive tasks and to reduce arousal and alertness.^18, 19, 20, 21, 22, 23, 24, 25, 26, 27, 28, 29^ Concomitant with these behavioral changes, SD increases functional magnetic resonance imaging (fMRI) signals in the thalamus, which is essential for alertness,^30^ while reducing fMRI signals in superior parietal (SPC) and prefrontal (PFC) cortices during a visual attention (VA) task.^30, 31^

The role of DA in the regulation of thalamic and PFC activity is well established.^32, 33^ For instance, D_2_/D_3_ receptors (D_2_/D_3_R) in the ventral striatum (VS) have been associated with fMRI activation of the medial PFC during visual attention to rewards,^34^ and D_2_/D_3_R in the dorsal striatum have been associated with neural processing in the PFC during inhibitory control^35^ and executive functioning.^36, 37^ However, the role of DA in the regulation of the SPC has not been investigated. Thus, while SD-related changes in the PFC and thalamic activation^30^ may have reflected the decreases in DA function during SD,^11, 13^ the association between the decreases in DA function and the changes in brain activation during SD are still largely unknown.

We recently showed that a balance between dorsal caudate versus VS in D_2_/D_3_R mediated the modulation of brain activation to a cognitive task.^38^ Thus, we predicted that fMRI signals during an attention task would show distinct linear associations with the dorsal and ventral striatal regions such that higher D_2_/D_3_R availability in the dorsal versus VS regions would be associated with greater cortical activation, and that SD would disrupt these associations.

Hence, in this work, we test the linear association between D_2_/D_3_R in the dorsal and ventral striatum and VA activation in thalamus, SPC and PFC, which are the three critical components of the attention networks.^2^ We measured D_2_/D_3_R using positron emission tomography (PET) and VA activation with 4-Tesla fMRI in 14 healthy men. Subjects were scanned with PET and fMRI twice, after one night of normal sleep (that is, under rested wakefulness (RW)) and also after one night of SD. We hypothesized that cortical activation responses would reflect the relative availability of D_2_/D_3_R in the dorsal (caudate, putamen) versus ventral striatum, whereas thalamic responses that are necessary for alertness^30^ would show an association with both dorsal and ventral striatum. We further predicted that SD would disrupt the modulation of striatal signaling in the indirect striatocortical pathway by virtue of the downregulation of striatal D_2_/D_3_ receptors that follows SD, which we have shown is associated with a concomitant impairment in cognitive performance.^11^

## Materials and methods

### Subjects

Fourteen healthy, non-smoking, right-handed men (age 32±8 years, education: 16±2 years) participated in the study. At *α*=0.05 and 80% power, this sample size allowed us to detect large effects (*r*=0.6) of SD on the association between D_2_/D_3_R and fMRI activation. The subjects were included if they were able to understand and give informed consent, and were 18 to 50 years old. They were screened carefully with a detailed medical history as well as physical and neurological examinations. The subjects were excluded if they had (1) urine positive for psychotropic drugs; (2) present or past history of dependence on alcohol or other drugs of abuse; (3) present or past history of neurological or psychiatric disorders (including sleep disorders); (4) cardiovascular disease or diabetes; (5) history of head trauma with loss of consciousness for more than 30 min; (6) medical conditions that may alter the brain function; (7) used psychoactive medications in the past month (that is, opiate analgesics, stimulants, sedatives); (8) used prescription (non-psychiatric) medication(s); or (9) contraindications to MRI environment (metallic implants/claustrophobia). The study participants signed a written consent approved by the Institutional Review Board at Brookhaven National Laboratory before the study. The subjects were asked to keep a diary of the number of hours slept per night for the 2-week duration of the study and this corresponded to an average of 7±1 h per night (range, 5–8 h).

### SD and RW sessions

**Figure 1:**
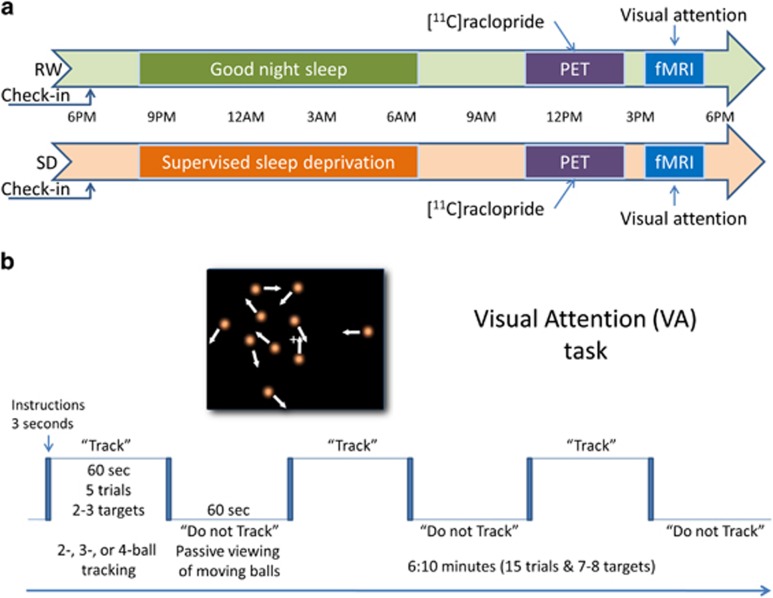
Study design. (**a**) Fourteen healthy, non-smoking, right-handed men were kept overnight onsite before their scheduled imaging sessions to ensure that that they had a good night rest (rested wakefulness (RW) session) or they did not sleep during the night (sleep deprivation (SD) session). All the subjects underwent [^11^C]raclopride positron emission tomography (PET) to assess D_2_/D_3_R in the striatum and 4-Tesla blood-oxygenation-level-dependent functional magnetic resonance imaging (BOLD-fMRI) to map brain activation to a visual attention (VA) task during RW and during SD. (**b**) The parametric VA task had a blocked design in which subjects either tracked 2, 3 or 4 balls out of 10 moving balls (task epochs) or viewed them passively (rest epochs).

All the subjects were kept overnight at the Brookhaven National Laboratory campus before their scheduled sessions (Figure 1a) to ensure that that they had a good night rest for the RW session (6.7±0.9 h of sleep; range 5–8.5 h) or they did not sleep during the night for the SD session (supervised by a team member). For the SD session, the total time of sleep deprivation, computed from the subject’s wake up time on the check-in day until the end of fMRI session, was 30–35 h. The SD and RW sessions were scheduled 2 weeks apart. The subjects did not have food after midnight and no caffeinated beverages were permitted during the study. PET and MRI acquisition were done sequentially on the same day, either after RW or SD. On the RW day, the subjects were awakened at 0700 h and brought to the imaging suite. A nurse remained with the subjects to ensure they stayed awake throughout the study. The PET sessions (RW and SD) took place between 1100 h and 1400 h and the MRI sessions (RW and SD) took place between 1500 h and 1700 h. Half the studies started with the RW session; the remaining studies started with the SD session to control for practice effects on brain activation.^39^

### PET imaging

A Siemens HR+ tomograph with 4.5 mm isotropic resolution was used to collect dynamic PET images in three-dimensional mode. Twenty emission scans were obtained from the time of injection up to 54 min immediately after injection of [^11^C]raclopride (4–8 mCi; specific activity 0.5–1.5 Ci μm^−1^). Arterial sampling was used to quantify total carbon-11 and unchanged [^11^C]raclopride in plasma. The distribution volume (DV) was computed for each imaging voxel using a graphical analysis technique for reversible systems.^40^ These images were then spatially normalized to the stereotactic space of the Montreal Neurological Institute using a 12-parameter affine transformation. A custom Montreal Neurological Institute template, which was previously developed using DV images acquired with [^11^C]raclopride and the same PET scanning sequence^41^ was used for the spatial normalization of the DV images. The intensity of the DV images was normalized to that in the cerebellum (left and right regions of interest) to quantify the non-displaceable binding potential (BP_ND_) in each voxel. BP_ND_ images were spatially smoothed (8-mm isotropic Gaussian kernel) using the statistical parametric mapping package SPM8 (Wellcome Trust Centre for Neuroimaging, London, UK).

### Anatomical region of interest analyses

In-house software written in IDL (Exelis Visual Information Solutions, Boulder, CO, USA) and the Automated Anatomical Labeling (AAL) atlas^42^ were used to define three bilateral anatomical regions of interest (ROIs): putamen (PU), caudate (CD) and VS (Figure 1a). The CD ROI included all voxels in dorsal caudate, as defined in the AAL atlas planes −6 mm<*z*<14 mm. The VS ROI encompassed the inferior planes of pre-commissural caudate and putamen (6 mm<*y*⩽27 mm; −11 mm<*z*⩽−6 mm) in the AAL atlas. Average BP_ND_ values were computed for each subject independently for these ROIs. We chose to report the average BPND values in the whole anatomy of the striatal regions to minimize human errors or potential confounds resulting from the utilization of arbitrary thresholds.

### VA paradigm

After the PET session, the subjects underwent fMRI with a VA task that was described previously.^39, 43, 44, 45, 46^ This fMRI task was used previously to assess visual attention activation in healthy controls,^39, 44, 47, 48, 49^ human immunodeficiency virus patients,^45, 50, 51, 52^ marijuana^53^ and cocaine^46, 54^ abusers as well as to assess the effects of functional connectivity,^54, 55^ sleep deprivation,^30^ dopamine transporters^56^ and stimulants^57^ on VA activation. The ball-tracking task activates attention-related brain regions (prefrontal, parietal, and occipital cortices, thalamus, and cerebellum). The blocked VA task had 3 difficulty levels (2-, 3, and 4-ball tracking). Each of the three fMRI runs lasted 6 min and was composed by three ‘TRACK’ epochs interleaved with three ‘DO NOT TRACK’ epochs. ‘TRACK’ epochs interleave five tracking and five respond periods (Figure 1b). In these epochs, a target set of balls (2, 3 or 4 out of 10 balls) is briefly highlighted. Then all the balls start to move. The subjects’ task is to fixate on the center cross and track the target balls as they move randomly (simulated Brownian motion) across the display with instantaneous angular speed of 3° per second. At the end of tracking periods, the balls stop moving and a new set of balls is highlighted; the subjects’ are instructed to press a button if the highlighted balls are the target set. After a 0.5-s delay, the original target balls are re-highlighted to re-focus the subjects’ attention on the target balls. ‘DO NOT TRACK’ epochs are composed of five consecutive ‘resting’ periods. In these epochs, all the 10 balls move and stop in the same manner as during ‘TRACK’ epochs; however, no balls are highlighted, and subjects are instructed to not track the balls and view them passively. The subjects performed a brief training session (~10 min) of a shortened version of the paradigm outside of the scanner to ensure that they understood and were able to perform the tasks. There were three fMRI runs (two-, three- and four-ball tracking). Each one of these runs had 231 image volumes (4 dummy volumes, 7 fixation cross baseline volumes, 112 passive-viewing volumes and 112 ball-tracking volumes).

Different versions of the two-, three- and four-ball-tracking tasks were used in each session (SD and RW). The stimuli were created using Matlab (MathWorks, Natick, MA, USA) and presented to the subjects on MRI-compatible goggles (Resonance Technology, Northridge, CA, USA) connected to a personal computer. The display software was synchronized with the MRI acquisition using a trigger pulse. All button press events were recorded to determine RT and performance accuracy during fMRI.

### MRI data acquisition

The blood-oxygenation-level-dependent (BOLD) contrast was used to assess fMRI activation in a 4-Tesla whole-body Varian/Siemens MRI scanner. A T2*-weighted single-shot gradient-echo planar imaging sequence (TE/TR=20/1600 ms, 4 mm slice thickness, 1 mm gap, 35 coronal slices, 3.1 mm in-plane resolution, 64 × 64 matrix size, 90°-flip angle, 231 time points, bandwidth: 200.00 kHz) covering the whole brain was used for this purpose. Padding was used to minimize motion. Task performance and subject motion were determined immediately after each fMRI trial.^58^ Anatomical images were collected using T2-weighted hyperecho (TE/TR=42/10 000 ms, echo train length=16, 256 × 256 matrix size, 30 coronal slices, 0.86 × 0.86 mm in-plane resolution, 5 mm thickness, 1 mm gap, 2-min scan time) and T1-weighted three-dimensional MDEFT (TE/TR=7/15ms, 0.94 × 0.94 × 1 mm spatial resolution, axial orientation, 256 readout and 192 × 96 phase-encoding steps, 16-min scan time) sequences. These structural MRI scans were reviewed to rule out gross morphological abnormalities in the brain.

### Data processing

The first four volumes in the time series were discarded to avoid non-equilibrium effects in the fMRI signal. Subsequent analyses were performed with SPM8. Spatial realignment was performed with a fourth degree B-spline function without weighting and without warping; head motion was less than 2-mm translations and 2° rotations for all scans. Spatial normalization to the stereotactic space of the Montreal Neurological Institute was performed using a 12-parameter affine transformation with medium regularization, 16-nonlinear iterations, 3 × 3 × 3 mm^3^ voxel size and the standard SPM8 EPI template. Spatial smoothing was carried out using an 8-mm (full width at half maximum) Gaussian kernel. A general linear model^59^ was used to calculate the BOLD contrasts for each VA load condition (two, three and four balls), session (RW and SD) and subject. The blocked analysis was based on a box-car design defined by the onsets of the ‘TRACK’ epochs, convolved with the canonical hemodynamic response function, as a low-pass filter, and a high-pass filter (256 s time cutoff).

### Statistical analyses

Simple (SLR) and multiple (MLR) linear regression analyses were used to assess the association between the fMRI signals in the brain and the D_2_/D_3_R measures across subjects, using VA load and session as covariates in SPM8. Five SLR models were used with regressors that reflected the absolute BP_ND_ values extracted from CD (SLR1), PU (SLR2) and VS (SLR3), as well as the relative BP_ND_ measures CD/VS (SLR4) and PU/VS (SLR5). Two different MLR models were used to study the combined influence of receptors in VS and in CD (MLR1), as well as that of receptors in PU and VS (MLR2). Specifically, the fMRI responses at a given voxel, S(*x, y, z*), were modeled using the affine transformation:



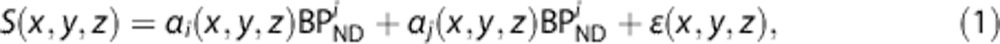



where *i* and *j* are CD and VS, or PU and VS, the scalar maps *α* (*x, y, z*) are the slopes that quantify the efficiency of the linear association between D_2_/D_3_R and brain activation and *ε* is the intercept of the MLR. Independent MLR analyses were carried for RW and SD as well as for the combined RW and SD sample. For all analyses, statistical significance was set as *P*_FWE_<0.05, corrected for multiple comparisons in the whole brain with the random field theory and a family-wise error correction at the cluster level. A cluster-forming threshold *P<*0.001 (two-sided) and a minimum cluster size of 100 voxels were used for this purpose.

## Results

### Behavior

The fMRI and behavioral data in this work were previously reported in a study that documented SD-related decreases in VA performance and fMRI activation differences between RW and SD.^30^ Briefly, subjects reported higher sleepiness before the SD session than before the RW session (RW: 3.8±0.5; s.d.: 8.8±0.4; *P<*0.0001, paired *t*-test). Increased sleepiness correlated linearly with performance accuracy during the fMRI tasks (*R*=0.59; *P=*0.025). Performance accuracy during fMRI decreased with increased task difficulty (from two balls to four balls; *P<*0.0001; two-way ANOVA) and was lower during the SD session than during the RW session (*P=*0.02). Reaction time (RT) during the fMRI did not differ significantly across tasks or sessions. There were no statistically significant load × session interaction effects on subject’s performance (accuracy or RT). In the present study, we studied the association between brain activation during the VA task and D_2_/D_3_R measures in the dorsal and ventral striatum.

### D_2_/D_3_R

**Figure 2:**
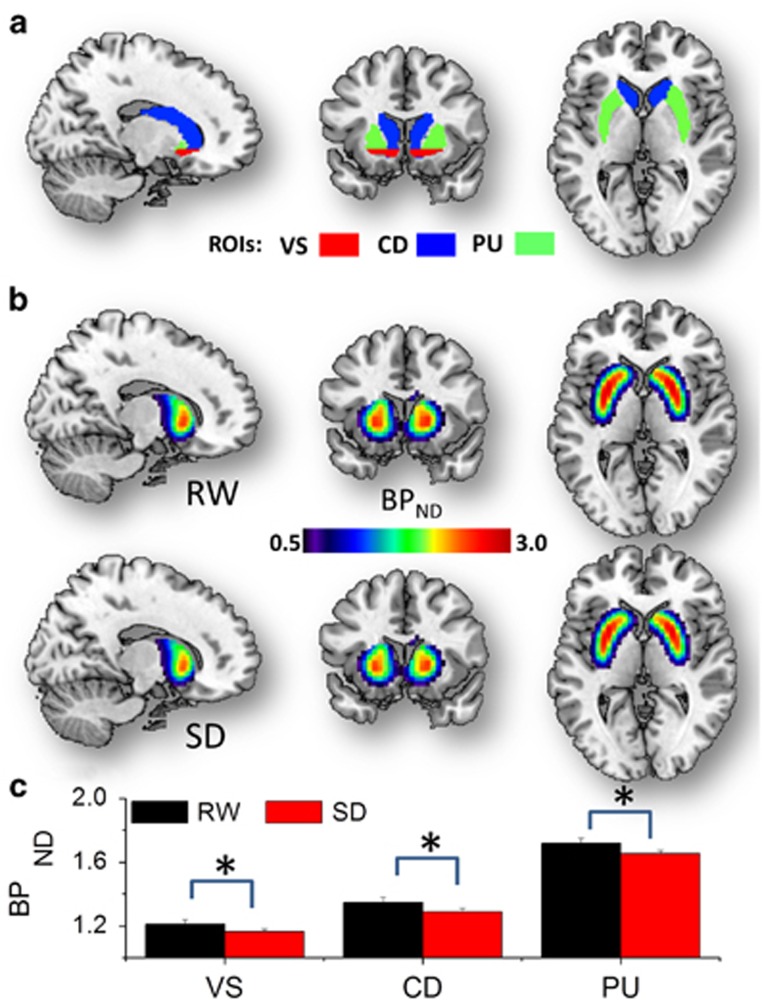
D2/D3R binding. (**a**) Average non-displaceable binding potential (BP_ND_) values reflecting D_2_/D_3_R levels were computed in three bilateral anatomical striatal regions of interest (ROIs): ventral striatum (VS), dorsal caudate (CD) and putamen (PU), superimposed on three orthogonal views of the human brain. (**b**) Average BP_ND_ maps across subjects for the sleep deprivation (SD) and rested wakefulness (RW) conditions, highlighting the high availability of D_2_/D_3_R in the striatum. (**c**) Bar plot quantifying the average BP_ND_ measures in the ROIs for RW and SD and highlighting the significantly lower availability of D_2_/D_3_R for SD than for RW (*P<0.05, two-sided). Sample size: 14 healthy, non-smoking, right-handed men. Error bars are s.e.m.

The average BP_ND_ values, which were computed without BP_ND_ thresholds over the anatomical volumes of CD, PU and VS (see the 'Methods' section), were lower for SD than for RW for all striatal ROIs (VS: 1.21±0.03 (RW) and 1.16±0.02 (s.d.); CD: 1.35±0.03 (RW) and 1.29±0.02 (s.d.); PU: 1.72±0.03 (RW) and 1.65±0.02 (s.d.); mean±s.e.; *P<*0.05, two-sided paired *t*-test, df=13; Figure 2). The BP_ND_ ROI measures showed high correlations across subjects and were higher during RW than during s.d. (*P<*0.05). The differences in the ‘relative’ BP_ND_ measures between RW and s.d. were not significant (CD/VS: 1.11±0.01 (RW) and 1.11±0.01 (s.d.); PU/VS: 1.42±0.01 (RW) and 1.42±0.01 (s.d.); *P*>0.2, two-sided paired *t*-test, df=13).

### D_2_/D_3_R and brain activation

**Figure 3:**
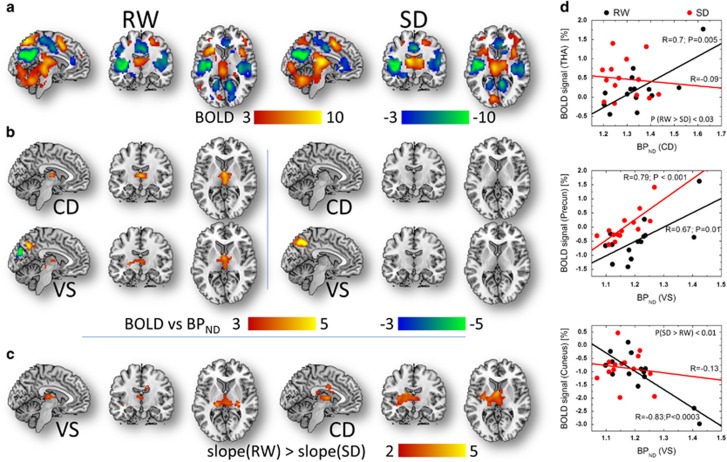
Visual attention activation versus dopamine (DA) receptors. Statistical significance (*t*-score) maps of brain activation responses for (**a**) rested wakefulness (RW) and for sleep deprivation (SD) conditions superimposed on three orthogonal views of the human brain (*P*_FWE_<0.0001) and (**b**) simple linear regression (SLR) slopes demonstrating the linear association across subjects between brain activation responses and D_2_/D_3_R separately for caudate (CD) and ventral striatum (VS; *P*_FWE_<0.001). (**c**) For VS and CD, the SLR slopes in the thalamus were significantly steeper for RW than for SD (*P*_FWE_<0.02). (**d**) Scatter plots showing the linear associations between D_2_/D_3_R measures in caudate (CD) and ventral striatum (VS), and the blood-oxygen-level dependent (BOLD) signals in thalamus, precuneus and cuneus, independently for the rested wakefulness (RW) and sleep deprivation (CD) conditions. Sample size: 14 healthy, non-smoking, right-handed men. FWE, family-wise error.

The SLR analysis revealed that fMRI signals in the thalamus increased linearly with D_2_/D_3_R across subjects during RW but not during SD, independently for CD, VS and PU (*P*_FWE_<0.003; Figure 3b and Table 1). The slopes of the linear associations between fMRI signals in the anterior thalamus and D_2_/D_3_R in the CD, and between fMRI signals in the posterior thalamus and D_2_/D_3_R in the VS were significantly steeper for RW than for SD (*P*_FWE_<0.02; Figure 3c and Table 1). During RW, higher availability of D_2_/D_3_R in the VS were associated with increased activation in precuneus and increased deactivation in cuneus; during SD only the fMRI signals in precuneus showed a linear association with D_2_/D_3_R in VS (*P*_FWE_<0.001; Figure 3b and Table 1). Figure 3d exemplifies the linear associations between D_2_/D_3_R measures in the striatum and fMRI signals in the thalamus, precuneus and cuneus, independently for RW and for SD.

### Balanced influence of D2/D3R in dorsal versus ventral striatum on fMRI signals

**Figure 4:**
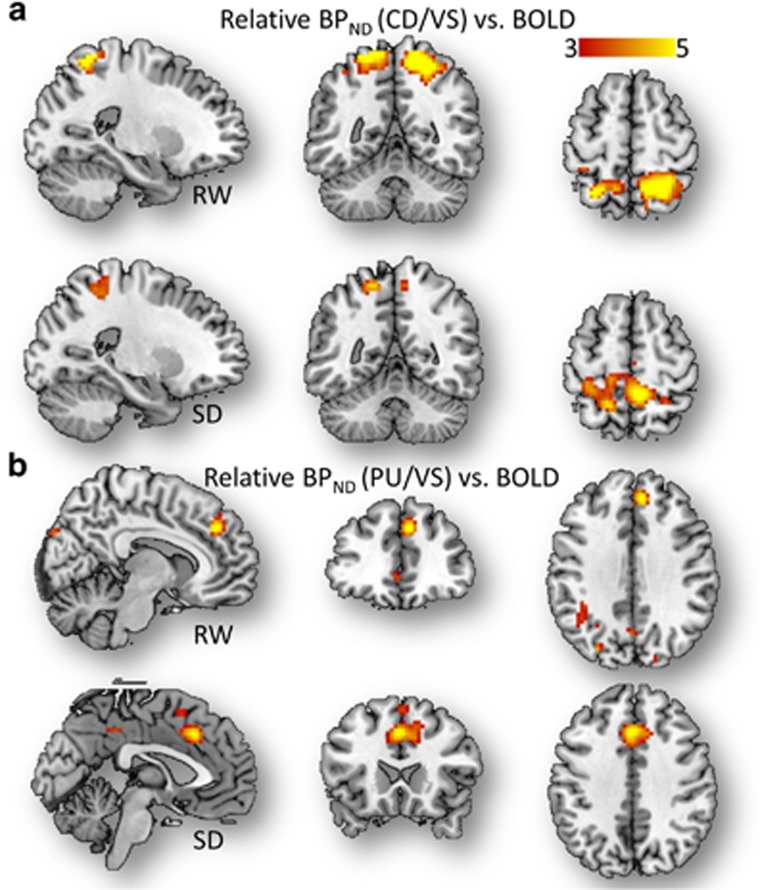
Parietal activation versus relative D_2_/D_3_R in dorsal to ventral striatum. (**a** and **b**) Statistical significance (*t*-score) maps for simple linear regression (SLR) slopes demonstrating the linear association across subjects between brain activation responses and the caudate (CD) to ventral striatum (VS) (**a**) and putamen (PU) to VS (**b**) ratios of D_2_/D_3_R measures for rested wakefulness (RW) and for sleep deprivation (SD), superimposed on three orthogonal views of the human brain. Sample size: 14 healthy, non-smoking, right-handed men. Significance threshold: *P*_FWE_<0.002, cluster corrected for multiple comparisons in the whole brain. BOLD, blood-oxygen-level dependent; FWE, family-wise error.

The SLR analysis also revealed significant linear associations between the ‘relative’ CD-to-VS ratio of D_2_/D_3_R measures and the fMRI signals in SPC (positive slope), regions that showed prominent brain activation to the VA task during RW but attenuated activation during SD (Table 2), and in precuneus (negative slope), a region that showed significant fMRI deactivation (negative BOLD signals) during the VA tasks, independently for RW and for SD (*P*_FWE_<0.03, cluster corrected for multiple comparisons in the whole brain; Figure 4 and Table 2).

**Figure 5:**
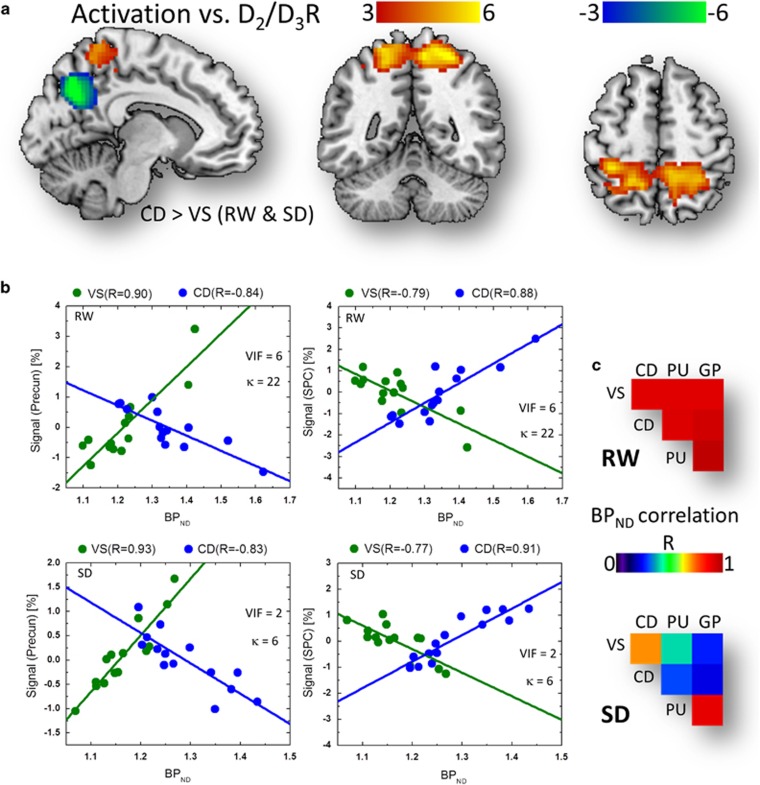
Balanced dopaminergic (DAergic) effects on parietal activation. (**a**) Statistical significance (*t*-score) maps for multiple linear regression (MLR) slopes demonstrating the linear associations across subjects between average non-displaceable binding potential (BP_ND_) measures in caudate (CD) and ventral striatum (VS) and brain activation responses in the superior parietal cortex (SPC; red-yellow pattern) and precuneus (blue-green pattern) during visual attention for rested wakefulness (RW) and for sleep deprivation (SD; conjunction analysis), superimposed on three orthogonal views of the human brain. Significance threshold: *P*_FWE_<0.002, cluster corrected for multiple comparisons in the whole brain. (**b**) Scatter plots showing the linear associations between the predicted signals (BP_ND_^VS^ and BP_ND_^CD^; see the 'Methods' section) in SPC and precuneus and the corresponding BP_ND_ measures in CD and VS. (**c**) BP_ND_ correlation matrix showing the Pearson correlation factors (R; computed across subjects) between average D_2_/D_3_R measures in VS, CD, putamen (PU) and globus pallidus (GP), for RW and for SD conditions. Sample size: 14 healthy, non-smoking, right-handed men. FWE, family-wise error; κ, condition number; VIF, variance inflation factor.

The MLR analysis showed a bilinear association between brain activation responses in parietal cortex and D_2_/D_3_R in VS and in CD (Figure 5a). Specifically, in precuneus, the fMRI responses predicted by D_2_/D_3_R in VS showed a positive correlation with BP_ND_^VS^, whereas those predicted by D_2_/D_3_R in CD showed a negative correlation with BP_ND_^CD^ (*P*_FWE_<0.0005, cluster corrected for multiple comparisons in the whole brain; RW and SD conjunction contrast), and the MLR slope was significantly steeper for VS than for CD (*α*_VS_>*α*_CD_, *P*_FWE_<0.0005; Figure 5b). Conversely, the predicted responses in SPC showed negative correlation with BP_ND_^VS^ and positive correlation with BP_ND_^CD^ (*P*_FWE_<0.0005), and the MLR slope was significantly steeper for CD than for VS (*α*_CD_>*α*_VS_, *P*_FWE_<0.002; Figure 5b). Although the SLR association between the relative CD-to-VS D_2_/D_3_R measures and the fMRI signals accounted for less than 22% of the variance in the fMRI data, the MLR association accounted for more than 52% of the variance in the fMRI signal in SPC and precuneus. However, because the BP_ND_^CD^ and BP_ND_^VS^ regressors exhibited high correlation (*R*=0.91 for RW and 0.71 for SD; Figure 5c), we evaluated the risk of multicollinearity in the MLR model using the variance inflation factor, VIF=1/(1−*R*^2^), and the condition number, *κ*=|*λ*_max_/*λ*_min_|, a standard measure reflecting the ratio between the maximum and minimum eigenvalues, *λ*, of the correlation matrix computed from BP_ND_^VS^ and BP_ND_^CD^. Depending on *κ* and VIF, the significance of the multicollinearity problem is usually classified as low (*κ*<30, VIF<10) or high (*κ*>30, VIF>10).^60, 61^ In the present work, the risk of multicollinearity for the BP_ND_^CD^ and BP_ND_^VS^ regressors did not exceed these thresholds for any of the sessions and was lower for SD (*κ*=6 and VIF=2) than for RW (*κ*=28 and VIF=6).

The fMRI responses in SMA, a PFC region that was increasingly activated by parametric VA load increases (BOLD signal=0.52±0.07% load effect=0.16%±0.10% mean±90% confidence interval; Table 2) and in ACC increased in proportion to the ‘relative’ PU-to-VS ratio (PU/VS) of BP_ND_ measures. Visual cortex deactivation was enhanced by VA load increases and attenuated by SD, and decreased in proportion to the relative PU-to-VS ratio of BP_ND_ measures during RW (*P*_FWE_<0.005; Figure 4 and Table 2). Similarly during SD, ACC activation showed a negative association with the PU-to-VS ratio of BP_ND_ measures (Table 1; *P*_FWE_<0.001).

**Figure 6:**
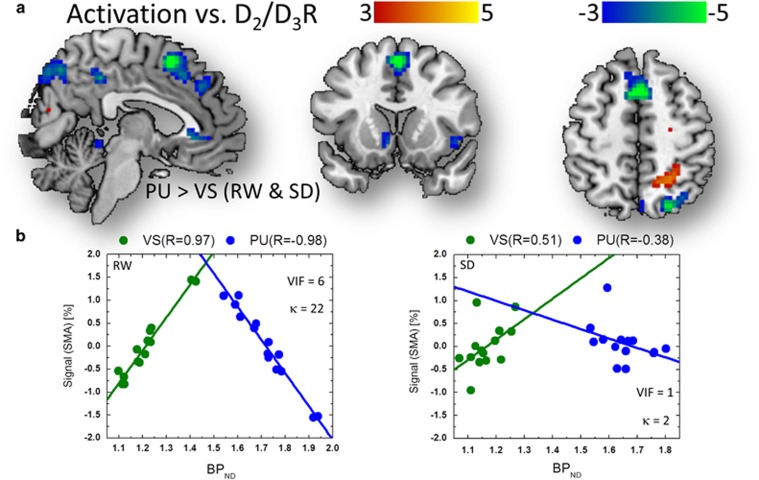
Balanced dopaminergic (DAergic) effects on prefrontal activation. (**a**) Statistical significance (*t*-score) maps for multiple regression analysis (MLR) slopes demonstrating the negative linear associations across subjects between average non-displaceable binding potential (BP_ND_) measures in putamen (PU) and ventral striatum (VS) and brain activation responses in the supplementary motor area (SMA; blue-green pattern) during visual attention for rested wakefulness (RW) and for sleep deprivation (SD; conjunction analysis), superimposed on three orthogonal views of the human brain. Significance threshold: *P*_FWE_<0.005, cluster corrected for multiple comparisons in the whole brain. (**b**) Scatter plots showing the linear associations between the predicted signals (BP_ND_^VS^ and BP_ND_^PU^; see the 'Methods' section) in SMA and the corresponding BP_ND_ measures in PU and VS.

The MLR analysis confirmed the bilinear association between brain activation responses and D_2_/D_3_R in VS and PU during RW and SD (Figure 6a). Specifically, in SMA, the fMRI responses predicted by D_2_/D_3_R in VS showed a positive linear association with BP_ND_^VS^, whereas those predicted by D_2_/D_3_R in PU showed a negative linear association with BP_ND_^PU^ (*P*_FWE_<0.03; RW and SD conjunction contrast), and the MLR slope was significantly steeper for VS than for PU (*α*_VS_>*α*_PU_, *P*_FWE_<0.005; Figure 6b). In cuneus, the fMRI responses predicted by D_2_/D_3_R in PU showed a positive correlation with BP_ND_^PU^, whereas those predicted by D_2_/D_3_R in VS showed a negative correlation with BP_ND_^VS^ (*P*_FWE_<0.001; RW and SD conjunction contrast), and the MLR slope was significantly steeper for PU than for VS (*α*_PU_>*α*_VS_, *P*_FWE_<0.005). The SLR association accounted for 38% of the variance in the fMRI data in SMA during RW (27% during SD). The MLR association accounted for 52% of the variance in the fMRI signal in SMA during RW (27% during SD). The risk of multicollinearity for the BP_ND_^PU^ and BP_ND_^VS^ regressors was lower for SD (*κ*=2 and VIF=1) than for RW (*κ*=22 and VIF=6).

### Sleep-deprivation effects: behavior vs brain activation

Across all ball-tracking conditions, SD-related decreases in performance accuracy were linearly associated with SD-related decreases in VA activation in the PFC (BA=24; *R*=0.52; *P<*0.0004; linear regression, df=41).

## Discussion

Here we demonstrate a distinct involvement of D_2_/D_3_R in the different striatal regions in the fMRI activation of brain regions involved in the alerting, orienting and executive components of attention^2^ during the VA task. We found that D_2_/D_3_R in dorsal striatum counterbalance D_2_/D_3_R in ventral striatum in the modulation of activation responses to a VA task, which corroborates our previous findings using a sensorimotor reaction time task.^38^ We also found that the SD-related reduction in the availability of D_2_/D_3_R in the striatum was associated with (1) decreased strength in the linear association between thalamic activation and D_2_/D_3_R in CD, PU and VS during SD and (2) a robust bilinear association between the activation of frontal and parietal regions and D_2_/D_3_R in dorsal relative to ventral striatal regions that attenuated the effects of SD. This study also documents a counterbalanced association between caudate versus VS D_2_/D_3_R in the deactivation of the default-mode network during VA.

### Thalamus

The thalamus, the gateway to the cortex,^62^ is essential for alerting attention^2^ and for arousal^63^ and has an important role in the regulation of sleep and wakefulness.^64^ Here we show for the first time the role of D_2_/D_3_R-mediated dopamine signaling in the activation of the thalamus. Specifically, thalamic activation increased in proportion to D_2_/D_3_R in the striatum during the RW condition but not during the SD condition, when D_2_/D_3_R availability was significantly reduced and thalamic activation was higher than for the RW condition. As the thalamus mediates the interaction between attention and arousal in humans^63^ and is involved in the alerting component of attention,^2, 65, 66^ the increased thalamic activation^14, 15, 16, 17, 30, 67^ likely reflects an adaptation to compensate for reduced DAergic signaling due to lower D_2_/D_3_R during SD. Previous studies have documented associations between striatal D_2_/D_3_R and cortical fMRI responses to emotion, visual attention, decision-making and inhibitory control tasks.^34, 35, 68, 69, 70^ These studies, however, did not report an association between D_2_/D_3_R and fMRI signals in the thalamus. Dopamine is a neuromodulator that changes the efficacy of other neurotransmitters as a function of ongoing neuronal activity.^71^ The effect of DA on neuronal firing is believed to improve signal to noise for the detection of task-specific neuronal activation in electrophysiological studies.^72, 73^ Thus, by decreasing non-task-related activity, DA stimulation increases efficiency and results in lower activation of task-specific regions.^72^ Therefore, the higher thalamic activation for SD than for RW is consistent with decreased efficiency due to lower DAergic signaling during SD. Alternatively it could also reflect an increased modulation by noradrenergic signaling as SD also disrupt noradrenergic activity.^74^

### SPC

The SPC is essential for orienting attention^2, 75^ and projects to multiple cortical and subcortical areas (including thalamus) and is engaged in cognitive operations such as selective attention and top-down control of attention.^31, 76, 77, 78, 79, 80, 81, 82, 83, 84^ Here we show that the fMRI signals in SPC increased in proportion to the relative availability of D_2_/D_3_R in CD to that in VS such that the higher the CD-to-VS ratio of D_2_/D_3_R, the higher the activation in SPC. The SPC, which is consistently activated by the VA task,^39, 43, 44, 46, 48, 85^ showed lower fMRI activation during SD than during RW.^30^ However, significant differences between RW and SD in the linear association of SPC activation and striatal D_2_/D_3_R were not found. Thus, the lower cortical activation for SD than for RW commonly reported in neuroimaging studies^14, 15, 16, 17, 30, 67, 86, 87, 88, 89, 90^ likely reflects effects of SD on other neurotransmitter systems (that is, cholinergic or noradrenergic).

The MLR findings suggest that D_2_/D_3_R in CD and VS have distinct roles in the modulation of SPC responses during VA. Indeed, the association between D_2_/D_3_R and fMRI signals in SPC was significantly stronger when two regressors (BP_ND_^VS^ and BP_ND_^CD^; *R*^2^=0.52) were used in the MLR model, compared with one regressor (BP_ND_^VS^/BP_ND_^CD^; *R*^2^=0.22). This finding supports the existence of a balanced D_2_/D_3_R modulation of cortical activation responses from CD and VS, which is consistent with our recent findings using a sensorimotor reaction time task in a different sample of healthy subjects.^38^ The reproducibility of the MLR findings across the RW and SD conditions strongly supports the existence of a balanced D_2_/D_3_R modulation between CD and VS for the SPC activation to a VA task that is robust to the SD challenge.

### SMA and ACC

The ACC and PFC have been implicated in the executive component of attention^2, 75^ and are involved in target detection and awareness.^91^ We found an association between the relative availability of D_2_/D_3_R in the striatum and the fMRI signals in ACC and SMA, such that increased D_2_/D_3_R in VS proportionally increased the fMRI signal in ACC/SMA and increased D_2_/D_3_R in PU proportionally decreased it. These findings are consistent with the well-established role of DA on executive function in the human brain,^92^ including its role in response control.^93^ DA modulation in ACC is important for executive function,^94, 95^ and DA modulation in SMA is important for response inhibition and response initiation.^93, 96, 97^ Though most studies on the DAergic modulation of executive function identify the CD as the striatal region that mediates this effect,^98, 99, 100^ others implicate the PU.^101, 102, 103^ Our findings suggest that during the VA task, DA modulates executive attention through counterbalanced D_2_/D_3_R signaling from PU and VS. Interestingly, fMRI activation in SMA and ACC and its association with D_2_/D_3_R did not differ for SD and RW, providing support for a robust and balanced DAergic modulation of executive attention.

### Precuneus

The fMRI signals in the ventral anterior precuneus showed linear association with the ‘relative’ availability of D_2_/D_3_R in CD and VS such that the higher the CD-to-VS ratio of D_2_/D_3_R, the greater the deactivation in precuneus, both during RW and during SD. The MLR findings suggest that D_2_/D_3_R in CD and VS mediate a balanced modulation of deactivation in precuneus, which is reproducible across sessions and robust to the SD challenge. This is consistent with the role of DA in the modulation of the precuneus,^56, 104^ a major hub in the default-mode network^105, 106^ that deactivates during the VA task.^47^ Note that a recent study on functional subdivisions of the precuneus revealed that ventral anterior precuneus, but not the dorsal precuneus, is connected to the default-mode network.^107^ This major association area has reciprocal connections with superior and inferior parietal, prefrontal, and occipital cortices as well as subcortical regions,^108^ including the thalamus.^109^ The precuneus, is also involved in alertness^110^ and activates during spatial^43, 47, 111^ and orienting^79, 112^ attention. Because DA innervation in the parietal cortex is scarce,^113, 114^ the association between D_2_/D_3_R documented here suggests indirect DA modulation through thalamo–cortical pathways rather than a direct modulation. The enhanced deactivation of the precuneus in subjects with higher CD-to-VS ratio of D_2_/D_3_R could reflect regulation of CD in orienting attention by facilitating attention processing while inhibiting the posterior default-mode network.

We have shown that SD decreases the specific binding of [^11^C]raclopride (measured as reduced D_2_/D_3_ receptor availability in striatum), which we initially interpreted to reflect increased competition for binding secondary to an increase in DA release during SD.^11^ However, a follow-up study showed that the changes in DA triggered by the stimulant drug methylphenidate were not affected by SD, which was a finding not consistent with SD increasing DA release.^13^ Moreover this was supported by microdialysis experiments in which we showed that SD did not increase DA release.^13^ This led us to conclude that the decreases in [^11^C]raclopride’s specific binding reflected a downregulation of D_2_/D_3_ receptors in striatum by SD. Though the mechanisms underlying the D_2_/D_3_ receptor downregulation by SD are unclear, we speculated that increases in adenosine following SD mediate the internalization of D_2_/D_3_ receptors.^115, 116^ Indeed, we subsequently showed that caffeine, which is an adenosine antagonist led to an increase in D_2_/D_3_ receptors in striatum, presumably by interfering with adenosine-mediated internalization of D_2_/D_3_ receptors.^117^ Regardless of the mechanism, what our current findings are showing is that despite the overall reductions in striatal D_2_/D_3_ receptors with SD the activation/deactivation in ACC, SMA, SPC and precuneus to VA is buffered by the counterbalanced modulation of D_2_/D_3_ receptor signaling in the dorsal relative to the VS through the indirect striatocortical pathway.

### Limitations

The multicollinearity of the D_2_/D_3_R regressors limits the generalizability of our approach. As the multicollinearity problem increases, the regression model estimates become unstable and their standard errors might get inflated. As multicollinearity is considered a potential concern only if VIF>10 or *κ*>30,^60, 61^ the MLR model for the RW condition (VIF=6 and *κ*=28) was deemed viable. Furthermore, similar MLR patterns were observed for the SD condition that had significantly lower multicollinearity risk (VIF<2 and *κ*<6) than the RW condition, demonstrating the reproducibility of the MLR findings. Also we ascribe a modulatory role to D_2_/D_3_R on the activation responses to the VA task on the basis of finding significant associations, but future studies that vary the levels of DA signaling are needed to confirm this. We cannot assess the influence of NA (noradrenaline) on VA activation. It is known that the DAergic circuits interact with NAergic circuits^118^ and that wakefulness-promoting medications such as modafinil may enhance arousal in humans by activation of the NAergic locus coeruleus.^119^ Thus, the SD-related activation changes may reflect NA changes to sustain arousal during SD.

In conclusion, our study documents a significant involvement of DA signaling through striatal D_2_/D_3_R in the orchestration of visual attention. SD disrupted DA’s regulation of the thalamus but not that of the SPC and PFC. Our findings also corroborate a balanced involvement of D_2_/D_3_R signaling in dorsal striatum (CD and PU) versus that in VS for the regulation of brain activation in regions involved in the VA task.

## Figures and Tables

**Table 1:** Statistical significance for the linear associations between striatal D_2_/D_3_R measures and brain activation responses (BOLD) during the visual attention (VA) task under sleep deprivation (SD) and rested wakefulness (RW) conditions

*Region*		*MNI coordinates (mm)*			*Brain activation*			*Session*	*D*_*2*_ */D*_*3*_ *R-BOLD SLR*			
										*Cluster level*		*Voxel level*	
*Name*	*BA/nucleus*	x	y	z	*VA, T*	*VA load, T*	*SD>RW, T*		P_*FWE-corr.*_	k	P_*FWE-corr.*_	T	
*Caudate (CD)*												
Thalamus	Anterior	0	−6	6	5.7	NS	NS	RW	0.001	220	<0.0005	4.5
Middle Occipital	19	−27	−84	24	−4.1	−1.7	NS	SD	0.023	109	0.006	−4.5
												
*Ventral striatum (VS)*												
Precuneus	7	3	−63	39	−7.0	NS	NS	RW	0.001	222	<0.0005	5.3
Thalamus	Anterior	0	−3	6	4.0	NS	NS	RW	0.003	179	0.001	4.2
Cuneus	18	6	−81	27	−12.0	−1.7	2.0	RW	0.03	101	0.008	−6.4
Precuneus	7	6	−54	45	NS	NS	2.2	SD	0.001	217	<0.0005	5.8
												
*Globus pallidus (GP)*												
Thalamus	Ventral posterior	24	−15	0	NS	NS	NS	RW	0	389	<0.0005	4.7
Precuneus	7	0	−63	36	−12.3	NS	NS	RW	0.004	170	0.001	4.7
Cuneus	18	6	−81	27	−12.0	−1.7	2.0	RW	0.031	101	0.008	−5.6
Middle Occipital	19	−27	−78	33	−9.0	−2.4	NS	RW	0.015	125	0.004	−5.6
Middle Occipital	39	42	−78	18	3.0	NS	NS	RW	0	283	<0.0005	−4.9
												
*Putamen (PU)*												
Thalamus	Ventral posterior	24	−12	0	NS	NS	1.7	RW	0	355	<0.0005	4.5
Middle Occipital	19	−27	−78	33	−9.0	−2.4	NS	RW	0.002	194	0.001	−6.7
Middle Occipital	39	42	−78	18	3.0	NS	NS	RW	0	340	<0.0005	−5.3
Lingual	37	24	−51	−9	−3.9	NS	NS	RW	0.005	164	0.001	−4.6
												
*CD*												
Thalamus	Pulvinar	18	−24	15	8.3	NS	2.8	RW>SD	0.02	430	0.001	5.0
												
*VS*												
Thalamus	Pulvinar	18	−24	18	7.1	NS	2.5	RW>SD	0.002	665	<0.0005	5.5

Abbreviations: BOLD, blood-oxygen-level dependent; FWE-corr., family-wise error corrected; NS, not significant; SLR, simple linear regression.

Sample size: 14 healthy non-smoking men.

**Table 2:** Statistical significance for the linear associations between relative striatal D_2_/D_3_R measures and brain activation responses (BOLD) during the visual attention (VA) task under sleep deprivation (SD) and rested wakefulness (RW) conditions

*Region*		*MNI coordinates (mm)*			*Brain activation*			*Session*	*Relative D*_*2*_ */D*_*3*_ *R-BOLD SLR*			
										*Cluster level*		*Voxel level*	
*Name*	*BA*	x	y	z	*VA, T*	*VA load, T*	*SD>RW, T*		P_*FWE-corr.*_	k	P_*FWE-corr.*_	T	
*Caudate-to-ventral striatum ratio (CD/VS)*												
Superior parietal	7	27	−57	63	14.9	NS	−3.1	RW	0.003	186	0.001	7.3
Superior parietal	5	−18	−51	66	4.5	NS	−2.4	RW	<0.0005	382	<0.0005	6.5
Precuneus	7	3	−66	39	−9.6	NS	−1.9	RW	0.028	103	0.007	−4.4
Precuneus	5	−6	−42	60	−6.4	2.3	NS	SD	<0.0005	514	<0.0005	5.7
Precuneus	7	9	−69	33	−14.0	1.7	NS	SD	0.007	148	0.002	5.4
												
*Globus pallidus-to-ventral striatum ratio (GP/VS)*												
Supramarginal	40	−57	−39	27	−5.5	−1.8	NS	RW	0.005	160	0.001	4.8
Cingulum	32	0	21	42	11.9	3.5	NS	SD	0.004	172	0.001	−5.8

*Putamen-to-ventral striatum ratio (PU/VS)*												
Lingual	18	−15	−87	−6	−2.4	NS	NS	RW	<0.0005	349	<0.0005	5.8
Calcarine	17	15	−60	15	−14.1	−3.1	3.0	RW	<0.0005	287	<0.0005	5.5
Cingulum	24	0	24	39	7.8	1.7	NS	SD	0.006	155	0.002	−5.5

Abbreviations: BOLD, blood-oxygen-level dependent; FWE-corr., family-wise error corrected; NS, not significant; SLR, simple linear regression.

Sample size: 14 healthy non-smoking men.

## References

[bib1] Lim J, Dinges D. Sleep deprivation and vigilant attention. Ann N Y Acad Sci 2008; 1129: 305–322. 18591490 10.1196/annals.1417.002

[bib2] Fan J, McCandliss B, Fossella J, Flombaum J, Posner M. The activation of attentional networks. Neuroimage 2005; 26: 471–479. 15907304 10.1016/j.neuroimage.2005.02.004

[bib3] Posner M, Rothbart M, Sheese B, Voelker P. Control networks and neuromodulators of early development. Dev Psychol 2012; 48: 827–835. 21942663 10.1037/a0025530 PMC3253251

[bib4] Coull J, AC N, Frith C. The noradrenergic alpha2 agonist clonidine modulates behavioural and neuroanatomical correlates of human attentional orienting and alerting. Cereb Cortex 2001; 11: 73–84. 11113036 10.1093/cercor/11.1.73

[bib5] Nieoullon A. Dopamine and the regulation of cognition and attention. Prog Neurobiol 2002; 67: 53–83. 12126656 10.1016/s0301-0082(02)00011-4

[bib6] Volkow N, Wang G, Fowler J, Logan J, Gerasimov M, Maynard L et al. Therapeutic doses of oral methylphenidate significantly increase extracellular dopamine in the human brain. J Neurosci 2001; 21: RC121. 11160455 10.1523/JNEUROSCI.21-02-j0001.2001 PMC6763805

[bib7] Cárdenas L, Houle S, Kapur S, Busto U. Oral D-amphetamine causes prolonged displacement of [11C]raclopride as measured by PET. Synapse 2004; 51: 27–31. 14579423 10.1002/syn.10282

[bib8] Volkow N, Fowler J, Logan J, Alexoff D, Zhu W, Telang F et al. Effects of modafinil on dopamine and dopamine transporters in the male human brain: clinical implications. JAMA 2009; 301: 1148–1154. 19293415 10.1001/jama.2009.351 PMC2696807

[bib9] Oken B, Salinsky M, Elsas S. Vigilance, alertness, or sustained attention: physiological basis and measurement. Clin Neurophysiol 2006; 119: 1885–1901. 10.1016/j.clinph.2006.01.017 PMC2865224 16581292

[bib10] Sugden C, Housden C, Aggarwal R, Sahakian B, Darzi A. Effect of pharmacological enhancement on the cognitive and clinical psychomotor performance of sleep-deprived doctors: a randomized controlled trial. Ann Surg 2012; 255: 222–227. 21997802 10.1097/SLA.0b013e3182306c99

[bib11] Volkow N, Wang G, Telang F, Fowler J, Logan J, Wong C et al. Sleep deprivation decreases binding of [11C]raclopride to dopamine D2/D3 receptors in the human brain. J Neurosci 2008; 28: 8454–8461. 18716203 10.1523/JNEUROSCI.1443-08.2008 PMC2710773

[bib12] Klumpers U, Veltman D, van Tol M, Kloet R, Boellaard R, Lammertsma A et al. Neurophysiological effects of sleep deprivation in healthy adults, a pilot study. PLoS One 2015; 10: e0116906. 25608023 10.1371/journal.pone.0116906 PMC4301911

[bib13] Volkow N, Tomasi D, Wang G, Telang F, Fowler J, Logan J et al. Evidence that sleep deprivation downregulates dopamine D2R in ventral striatum in the human brain. J Neurosci 2012; 32: 6711–6717. 22573693 10.1523/JNEUROSCI.0045-12.2012 PMC3433285

[bib14] Chee M, Tan J, Zheng H, Parimal S, Weissman D, Zagorodnov V et al. Lapsing during sleep deprivation is associated with distributed changes in brain activation. J Neurosci 2008; 28: 5519–5528. 18495886 10.1523/JNEUROSCI.0733-08.2008 PMC6670628

[bib15] Tucker A, Rakitin B, Basner R, Gazes Y, Steffener J, Stern Y. fMRI activation during failures to respond key to understanding performance changes with sleep deprivation. Behav Brain Res 2011; 218: 73–79. 21074577 10.1016/j.bbr.2010.11.012 PMC3022081

[bib16] Venkatraman V, Huettel S, Chuah L, Payne J, Chee M. Sleep deprivation biases the neural mechanisms underlying economic preferences. J Neurosci 2011; 31: 3712–3718. 21389226 10.1523/JNEUROSCI.4407-10.2011 PMC6622793

[bib17] Chee M, Chuah L, Venkatraman V, Chan W, Philip P, Dinges D. Functional imaging of working memory following normal sleep and after 24 and 35 h of sleep deprivation: correlations of fronto-parietal activation with performance. Neuroimage 2006; 31: 419–428. 16427321 10.1016/j.neuroimage.2005.12.001

[bib18] Wesensten N, Belenky G, Kautz M, Thorne D, Reichardt R, Balkin T. Maintaining alertness and performance during sleep deprivation: modafinil versus caffeine. Psychopharmacology (Berl) 2002; 159: 238–247. 11862356 10.1007/s002130100916

[bib19] Wesensten N, Killgore W, Balkin T. Performance and alertness effects of caffeine, dextroamphetamine, and modafinil during sleep deprivation. J Sleep Res 2005; 14: 255–266. 16120100 10.1111/j.1365-2869.2005.00468.x

[bib20] Pilcher J, Huffcutt A. Effects of sleep deprivation on performance: a meta-analysis. Sleep 1996; 19: 318–326. 8776790 10.1093/sleep/19.4.318

[bib21] Harrison Y, Horne J, Rothwell A. Prefrontal neuropsychological effects of sleep deprivation in young adults—a model for healthy aging? Sleep 2000; 23: 1067–1073. 11145321

[bib22] Harrison Y, Horne J. The impact of sleep deprivation on decision making: a review. J Exp Psychol Appl 2000; 6: 236–249. 11014055 10.1037//1076-898x.6.3.236

[bib23] Harrison Y, Horne J. Sleep loss and temporal memory. Q J Exp Psychol A 2000; 53: 271–279. 10718074 10.1080/713755870

[bib24] Nilsson J, Söderström M, Karlsson A, Lekander M, Akerstedt T, Lindroth N et al. Less effective executive functioning after one night's sleep deprivation. J Sleep Res 2005; 14: 1–6. 15743327 10.1111/j.1365-2869.2005.00442.x

[bib25] Hsieh S, Cheng I, Tsai L. Immediate error correction process following sleep deprivation. J Sleep Res 2007; 16: 137–147. 17542943 10.1111/j.1365-2869.2007.00583.x

[bib26] Tsai L, Young H, Hsieh S, Lee C. Impairment of error monitoring following sleep deprivation. Sleep 2005; 28: 707–713. 16477957 10.1093/sleep/28.6.707

[bib27] Jennings J, Monk T, van der Molen M. Sleep deprivation influences some but not all processes of supervisory attention. Psychol Sci 2003; 14: 473–479. 12930479 10.1111/1467-9280.02456

[bib28] Volkow N, Wang G, Hitzemann R, Fowler J, Pappas N, Lowrimore P et al. Depression of thalamic metabolism by lorazepam is associated with sleepiness. Neuropsychopharmacology 1995; 12: 123–132. 7779240 10.1016/0893-133X(94)00068-B

[bib29] Fiset P, Paus T, Daloze T, Plourde G, Meuret P, Bonhomme V et al. Brain mechanisms of propofol-induced loss of consciousness in humans: a positron emission tomographic study. J Neurosci 1999; 19: 5506–5513. 10377359 10.1523/JNEUROSCI.19-13-05506.1999 PMC6782309

[bib30] Tomasi D, Wang R, Telang F, Boronikolas V, Jayne M, Wang G et al. Impairment of attentional networks after 1 night of sleep deprivation. Cereb Cortex 2009; 19: 233–240. 18483003 10.1093/cercor/bhn073 PMC2638746

[bib31] Behrmann M, Geng J, Shomstein S. Parietal cortex and attention. Curr Opin Neurobiol 2004; 14: 212–217. 15082327 10.1016/j.conb.2004.03.012

[bib32] Graham GD, Howseman AM, Rothman DL, Lantos G, Fayad PB, Brass LM et al. Proton magnetic resonance spectroscopy of metabolites after cerebral infarction in humans. Stroke 1991; 22: 143.

[bib33] Posner M, Rothbart M. Toward a physical basis of attention and self regulation. Phys Life Rev 2009; 6: 103–120. 20161073 10.1016/j.plrev.2009.02.001 PMC2748943

[bib34] Asensio S, Romero M, Romero F, Wong C, Alia-Klein N, Tomasi D et al. Striatal dopamine D2 receptor availability predicts the thalamic and medial prefrontal responses to reward in cocaine abusers three years later. Synapse 2010; 64: 397–402. 20034014 10.1002/syn.20741 PMC2840182

[bib35] Ghahremani D, Lee B, Robertson C, Tabibnia G, Morgan A, De Shetler N et al. Striatal dopamine D_2_/D_3_ receptors mediate response inhibition and related activity in frontostriatal neural circuitry in humans. J Neurosci 2012; 32: 7316–7324. 22623677 10.1523/JNEUROSCI.4284-11.2012 PMC3517177

[bib36] Volkow ND, Fowler JS, Wang GJ, Hitzemann R, Logan J, Schlyer DJ et al. Decreased dopamine D2 receptor availability is associated with reduced frontal metabolism in cocaine abusers. Synapse 1993; 14: 169–177. 8101394 10.1002/syn.890140210

[bib37] Volkow N, Gur R, Wang G, Fowler J, Moberg P, Ding Y et al. Association between decline in brain dopamine activity with age and cognitive and motor impairment in healthy individuals. Am J Psychiatry 1998; 155: 344–349. 9501743 10.1176/ajp.155.3.344

[bib38] Tomasi D, Wang G, Volkow N. Balanced modulation of striatal activation from D2/D3 receptors in caudate and ventral striatum: disruption in cannabis abusers. Hum Brain Mapp 2015; 36: 3154–3166. 26058801 10.1002/hbm.22834 PMC6868956

[bib39] Tomasi D, Ernst T, Caparelli EC, Chang L. Practice-induced changes of brain function during visual attention: a parametric fMRI study at 4 Tesla. Neuroimage 2004; 23: 1414–1421. 15589105 10.1016/j.neuroimage.2004.07.065

[bib40] Logan J, Fowler J, Volkow N, Wolf A, Dewey S, Schlyer D et al. Graphical analysis of reversible radioligand binding from time-activity measurements applied to [N-11C-methyl]-(-)-cocaine PET studies in human subjects. J Cereb Blood Flow Metab 1990; 10: 740–747. 2384545 10.1038/jcbfm.1990.127

[bib41] Wang G, Smith L, Volkow N, Telang F, Logan J, Tomasi D et al. Decreased dopamine activity predicts relapse in methamphetamine abusers. Mol Psychiatry 2011; 17: 918–925. 21747399 10.1038/mp.2011.86 PMC3261322

[bib42] Tzourio-Mazoyer N, Landeau B, Papathanassiou D, Crivello F, Etard O, Delcroix N et al. Automated anatomical labeling of activations in SPM using a macroscopic anatomical parcellation of the MNI MRI single-subject brain. Neuroimage 2002; 15: 273–289. 11771995 10.1006/nimg.2001.0978

[bib43] Culham JC, Brandt SA, Cavanagh P, Kanwisher NG, Dale AM, Tootell RBH. Cortical fMRI activation produced by attentive tracking of moving targets. J Neurophysiol 1998; 80: 2657–2670. 9819271 10.1152/jn.1998.80.5.2657

[bib44] Jovicich J, Peters RJ, Koch C, Braun J, Chang L, Ernst T. Brain areas specific for attentional load in a motion tracking task. J Cogn Neurosci 2001; 13: 1048–1058. 11784443 10.1162/089892901753294347

[bib45] Chang L, Tomasi D, Yakupov R, Lozar C, Arnold S, Caparelli E et al. Adaptation of the attention network in human immunodeficiency virus brain injury. Ann Neurol 2004; 56: 259–272. 15293278 10.1002/ana.20190

[bib46] Tomasi D, Goldstein R, Telang F, Maloney T, Alia-Klein N, Caparelli E et al. Thalamo-cortical dysfunction in cocaine abusers: implications in attention and perception. Psych Res Neuroimaging 2007; 155: 189–201. 10.1016/j.pscychresns.2007.03.002 PMC2265105 17582746

[bib47] Tomasi D, Ernst T, Caparelli E, Chang L. Common deactivation patterns during working memory and visual attention tasks: an intra-subject fMRI study at 4 Tesla. Hum Brain Mapp 2006; 27: 694–705. 16404736 10.1002/hbm.20211 PMC2424317

[bib48] Tomasi D, Chang L, Caparelli E, Ernst T. Different activation patterns for working memory load and visual attention load. Brain Res 2007; 1132: 158–165. 17169343 10.1016/j.brainres.2006.11.030 PMC1831676

[bib49] Tomasi D, Chang L, Caparelli E, Ernst T. Sex differences in sensory gating of the thalamus during auditory interference of visual attention tasks. Neurosci 2008; 151: 1006–1015. 10.1016/j.neuroscience.2007.08.040 PMC2262922 18201838

[bib50] Ernst T, Yakupov R, Nakama H, Crocket G, Cole M, Watters M, Ricardo-Dukelow M et al. Declined neural efficiency in cognitively stable human immunodeficiency virus patients. Ann Neurol 2009; 65: 316–325. 19334060 10.1002/ana.21594 PMC2734503

[bib51] Chang L, Yakupov R, Nakama H, Stokes B, Ernst T. Antiretroviral treatment is associated with increased attentional load-dependent brain activation in HIV patients. J Neuroimmune Pharmacol 2008; 3: 95–104. 18247124 10.1007/s11481-007-9092-0 PMC2745242

[bib52] Chang L, Holt J, Yakupov R, Jiang C, Ernst T. Lower cognitive reserve in the aging human immunodeficiency virus-infected brain. Neurobiol Aging 2013; 34: 1240–1253. 23158761 10.1016/j.neurobiolaging.2012.10.012 PMC3984923

[bib53] Chang L, Yakupov R, Cloak C, Ernst T. Marijuana use is associated with a reorganized visual-attention network and cerebellar hypoactivation. Brain 2006; 129: 1096–1112. 16585053 10.1093/brain/awl064

[bib54] Tomasi D, Volkow N, Wang R, Carrillo J, Maloney T, Alia-Klein N et al. Disrupted functional connectivity with dopaminergic midbrain in cocaine abusers. PLoS One 2010; 5: e10815. 20520835 10.1371/journal.pone.0010815 PMC2876035

[bib55] Tomasi D, Wang R, Wang G, Volkow N. Functional connectivity and brain activation: a synergistic approach. Cereb Cortex 2013; 24: 2619–2629. 23645721 10.1093/cercor/bht119 PMC4229895

[bib56] Tomasi D, Volkow N, Wang R, Telang F, Wang G, Chang L et al. Dopamine transporters in striatum correlate with deactivation in the default mode network during visuospatial attention. PLoS One 2009; 4: e6102. 19564918 10.1371/journal.pone.0006102 PMC2699543

[bib57] Tomasi D, Volkow N, Wang G, Wang R, Telang F, Caparelli E et al. Methylphenidate enhances brain activation and deactivation responses to visual attention and working memory tasks in healthy controls. Neuroimage 2011; 54: 3101–3110. 21029780 10.1016/j.neuroimage.2010.10.060 PMC3020254

[bib58] Caparelli EC, Tomasi D, Arnold S, Chang L, Ernst T. k-Space based summary motion detection for functional magnetic resonance imaging. Neuroimage 2003; 20: 1411–1418. 14568510 10.1016/S1053-8119(03)00339-2

[bib59] Friston KJ, Holmes AP, Worsley KJ, Poline JB, Frith CD, Franckowiak RSJ. Statistical parametric maps in functional imaging: a general approach. Hum Brain Map 1995; 2: 189–210.

[bib60] O’Brien R. A caution regarding rules of thumb for variance inflation factors. Qual Quant 2007; 41: 673–690.

[bib61] Freud R, Littell R. SAS System for Regression, 3rd Edn. SAS Institute and John Wiley and Sons: Cary, NC, USA, 2003.

[bib62] McAlonan K, Cavanaugh J, Wurtz R. Guarding the gateway to cortex with attention in visual thalamus. Nature 2008; 456: 391–394. 18849967 10.1038/nature07382 PMC2713033

[bib63] Portas C, Rees G, Howseman A, Josephs O, Turner R, Frith C. A specific role for the thalamus in mediating the interaction of attention and arousal in humans. J Neurosci 1998; 18: 8979–8989. 9787003 10.1523/JNEUROSCI.18-21-08979.1998 PMC6793555

[bib64] Lemieux M, Chen J, Lonjers P, Bazhenov M, Timofeev I. The impact of cortical deafferentation on the neocortical slow oscillation. J Neurosci 2014; 34: 5689–5703. 24741059 10.1523/JNEUROSCI.1156-13.2014 PMC3988418

[bib65] Christian B, Lehrer D, Shi B, Narayanan T, Strohmeyer P, Buchsbaum M et al. Measuring dopamine neuromodulation in the thalamus: using [F-18]fallypride PET to study dopamine release during a spatial attention task. Neuroimage 2006; 31: 139–152. 16469510 10.1016/j.neuroimage.2005.11.052

[bib66] Vandewalle G, Balteau E, Phillips C, Degueldre C, Moreau V, Sterpenich V et al. Daytime light exposure dynamically enhances brain responses. Curr Biol 2006; 16: 1616–1621. 16920622 10.1016/j.cub.2006.06.031

[bib67] Ma N, Dinges D, Basner M, Rao H. How acute total sleep loss affects the attending brain: a meta-analysis of neuroimaging studies. Sleep 2015; 38: 233–240. 25409102 10.5665/sleep.4404 PMC4288604

[bib68] Kienast T, Siessmeier T, Wrase J, Braus D, Smolka M, Buchholz H et al. Ratio of dopamine synthesis capacity to D2 receptor availability in ventral striatum correlates with central processing of affective stimuli. Eur J Nucl Med Mol Imaging 2008; 35: 1147–1158. 18202844 10.1007/s00259-007-0683-z

[bib69] Volkow N, Tomasi D, Wang G, Telang F, Fowler J, Wang R et al. Hyperstimulation of striatal D2 receptors with sleep deprivation: implications for cognitive impairment. Neuroimage 2009; 45: 1232–1240. 19349237 10.1016/j.neuroimage.2009.01.003 PMC2714585

[bib70] Kohno M, Ghahremani D, Morales A, Robertson C, Ishibashi K, Morgan A et al. Risk-taking behavior: dopamine d2/d3 receptors, feedback, and frontolimbic activity. Cereb Cortex 2015; 25: 236–245. 23966584 10.1093/cercor/bht218 PMC4259280

[bib71] Kiyatkin E, Rebec G. Dopaminergic modulation of glutamate-induced excitations of neurons in the neostriatum and nucleus accumbens of awake, unrestrained rats. J Neurophysiol 1996; 75: 142–153. 8822548 10.1152/jn.1996.75.1.142

[bib72] Volkow N, Fowler J, Wang G, Telang F, Logan J, Wong C et al. Methylphenidate decreased the amount of glucose needed by the brain to perform a cognitive task. PLoS One 2008; 3: e2017. 18414677 10.1371/journal.pone.0002017 PMC2291196

[bib73] Rolls E, Thorpe S, Boytim M, Szabo I, Perrett D. Responses of striatal neurons in the behaving monkey. 3. Effects of iontophoretically applied dopamine on normal responsiveness. Neurosci 1984; 12: 1201–1212. 10.1016/0306-4522(84)90014-9 6148716

[bib74] Mallick B, Singh A. REM sleep loss increases brain excitability: role of noradrenaline and its mechanism of action. Sleep Med Rev 2011; 15: 165–178. 21482157 10.1016/j.smrv.2010.11.001

[bib75] Posner M, Walker J, Friedrich F, Rafal R. Effects of parietal injury on covert orienting of attention. J Neurosci 1984; 4: 1863–1874. 6737043 10.1523/JNEUROSCI.04-07-01863.1984 PMC6564871

[bib76] Corbetta M, Shulman G. Control of goal-directed and stimulus-driven attention in the brain. Nat Rev Neurosci 2002; 3: 201–215. 11994752 10.1038/nrn755

[bib77] Fassbender C, Murphy K, Foxe J, Wylie G, Javitt D, Robertson I et al. A topography of executive functions and their interactions revealed by functional magnetic resonance imaging. Brain Res Cogn Brain Res 2004; 20: 132–143. 15183386 10.1016/j.cogbrainres.2004.02.007

[bib78] Lawrence N, Ross T, Hoffmann R, Garavan H, Stein E. Multiple neuronal networks mediate sustained attention. J Cogn Neurosci 2003; 15: 1028–1038. 14614813 10.1162/089892903770007416

[bib79] Le T, Pardo J, Hu X. 4 T-fMRI study of nonspatial shifting of selective attention: cerebellar and parietal contributions. J Neurophysiol 1998; 79: 1535–1548. 9497430 10.1152/jn.1998.79.3.1535

[bib80] de Fockert J, Rees G, Frith C, Lavie N. The role of working memory in visual selective attention. Science 2001; 291: 1803–1806. 11230699 10.1126/science.1056496

[bib81] Leonards U, Sunaert S, Van Hecke P, Orban G. Attention mechanisms in visual search—an fMRI study. J Cogn Neurosci 2000; 12(Suppl 2): 61–75. 11506648 10.1162/089892900564073

[bib82] Adler C, Sax K, Holland S, Schmithorst V, Rosenberg L, Strakowski S. Changes in neuronal activation with increasing attention demand in healthy volunteers: an fMRI study. Synapse 2001; 42: 266–272. 11746725 10.1002/syn.1112

[bib83] Buchel C, Josephs O, Rees G, Turner R, Frith CD, Friston KJ. The functional anatomy of attention to visual motion: a functional MRI study. Brain 1998; 121: 1281–1294. 9679780 10.1093/brain/121.7.1281

[bib84] Arrington C, Carr T, Mayer A, Rao S. Neural mechanisms of visual attention: object-based selection of a region in space. J Cogn Neurosci 2000; 12(Suppl 2): 106–117. 11506651 10.1162/089892900563975

[bib85] Tomasi D, Caparelli EC, Chang L, Ernst T. fMRI-acoustic noise alters brain activation during working memory tasks. Neuroimage 2005; 27: 377–386. 15893942 10.1016/j.neuroimage.2005.04.010 PMC2449823

[bib86] Chee M, Choo W. Functional imaging of working memory after 24 hr of total sleep deprivation. J Neurosci 2004; 24: 4560–4567. 15140927 10.1523/JNEUROSCI.0007-04.2004 PMC6729385

[bib87] Chee M, Chuah Y. Functional neuroimaging and behavioral correlates of capacity decline in visual short-term memory after sleep deprivation. Proc Natl Acad Sci USA 2007; 104: 9487–9492. 17517619 10.1073/pnas.0610712104 PMC1874228

[bib88] Drummond S, Brown G, Gillin J, Stricker J, Wong E, Buxton R. Altered brain response to verbal learning following sleep deprivation. Nature 2000; 403: 655–657. 10688201 10.1038/35001068

[bib89] Drummond S, Brown G, Stricker J, Buxton R, Wong E, Gillin J. Sleep deprivation-induced reduction in cortical functional response to serial subtraction. Neuroreport 1999; 10: 3745–3748. 10716202 10.1097/00001756-199912160-00004

[bib90] Chuah Y, Venkatraman V, Dinges D, Chee M. The neural basis of interindividual variability in inhibitory efficiency after sleep deprivation. J Neurosci 2006; 26: 7156–7162. 16822972 10.1523/JNEUROSCI.0906-06.2006 PMC6673955

[bib91] Petersen S, Posner M. The attention system of the human brain: 20 years after. Annu Rev Neurosci 2012; 35: 73–89. 22524787 10.1146/annurev-neuro-062111-150525 PMC3413263

[bib92] Monchi O, Ko J, Strafella A. Striatal dopamine release during performance of executive functions: a [(11)C] raclopride PET study. Neuroimage 2006; 33: 907–912. 16982202 10.1016/j.neuroimage.2006.06.058 PMC2967527

[bib93] Bari A, Robbins T. Inhibition and impulsivity: behavioral and neural basis of response control. Prog Neurobiol 2013; 108: 44–79. 23856628 10.1016/j.pneurobio.2013.06.005

[bib94] Bush G, Luu P, Posner M. Cognitive and emotional influences in anterior cingulate cortex. Trends Cognit Sci 2000; 4: 215–222. 10827444 10.1016/s1364-6613(00)01483-2

[bib95] MacDonald Ar, Cohen J, Stenger V, Carter C. Dissociating the role of the dorsolateral prefrontal and anterior cingulate cortex in cognitive control. Science 2000; 288: 1835–1838. 10846167 10.1126/science.288.5472.1835

[bib96] Li C, Huang C, Constable R, Sinha R. Imaging response inhibition in a stop-signal task: neural correlates independent of signal monitoring and post-response processing. J Neurosci 2006; 26: 186–192. 16399686 10.1523/JNEUROSCI.3741-05.2006 PMC6674298

[bib97] Mostofsky S, Simmonds D. Response inhibition and response selection: two sides of the same coin. J Cogn Neurosci 2008; 20: 751–761. 18201122 10.1162/jocn.2008.20500

[bib98] Rinne J, Portin R, Ruottinen H, Nurmi E, Bergman J, Haaparanta M et al. Cognitive impairment and the brain dopaminergic system in Parkinson disease: [18 F]fluorodopa positron emission tomographic study. Arch Neurol 2000; 57: 470–475. 10768619 10.1001/archneur.57.4.470

[bib99] Marié R, Barré L, Dupuy B, Viader F, Defer G, Baron J. Relationships between striatal dopamine denervation and frontal executive tests in Parkinson's disease. Neurosci Lett 1999; 260: 77–80. 10025703 10.1016/s0304-3940(98)00928-8

[bib100] Jokinen P, Brück A, Aalto S, Forsback S, Parkkola R, Rinne J. Impaired cognitive performance in Parkinson's disease is related to caudate dopaminergic hypofunction and hippocampal atrophy. Parkinsonism Relat Disord 2009; 15: 88–93. 18434233 10.1016/j.parkreldis.2008.03.005

[bib101] Müller U, Wächter T, Barthel H, Reuter M, von Cramon D. Striatal [123I]beta-CIT SPECT and prefrontal cognitive functions in Parkinson's disease. J Neural Transm 2000; 107: 303–319. 10821439 10.1007/s007020050025

[bib102] Cropley V, Fujita M, Bara-Jimenez W, Brown A, Zhang X, Sangare J et al. Pre- and post-synaptic dopamine imaging and its relation with frontostriatal cognitive function in Parkinson disease: PET studies with [11C]NNC 112 and [18 F]FDOPA. Psychiatry Res 2008; 163: 171–182. 18504119 10.1016/j.pscychresns.2007.11.003

[bib103] Siepel F, Brønnick K, Booij J, Ravina B, Lebedev A, Pereira J et al. Cognitive executive impairment and dopaminergic deficits in *de novo* Parkinson's disease. Mov Disord 2014; 29: 1802–1808. 25284687 10.1002/mds.26051

[bib104] Braskie M, Landau S, Wilcox C, Taylor S, O'Neil J, Baker S et al. Correlations of striatal dopamine synthesis with default network deactivations during working memory in younger adults. Hum Brain Mapp 2011; 32: 947–961. 20578173 10.1002/hbm.21081 PMC3176660

[bib105] Tomasi D, Volkow N. Functional connectivity density mapping. Proc Natl Acad Sci USA 2010; 107: 9885–9890. 20457896 10.1073/pnas.1001414107 PMC2906909

[bib106] Tomasi D, Volkow N. Association between functional connectivity hubs and brain networks. Cereb Cortex 2011; 21: 2003–2013. 21282318 10.1093/cercor/bhq268 PMC3165965

[bib107] Zhang S, Li C. Functional connectivity mapping of the human precuneus by resting state fMRI. Neuroimage 2012; 59: 3548–3562. 22116037 10.1016/j.neuroimage.2011.11.023 PMC3288461

[bib108] Cavanna A, Trimble M. The precuneus: a review of its functional anatomy and behavioural correlates. Brain 2006; 129: 564–583. 16399806 10.1093/brain/awl004

[bib109] Fernández-Espejo D, Soddu A, Cruse D, Palacios E, Junque C, Vanhaudenhuyse A et al. A role for the default mode network in the bases of disorders of consciousness. Ann Neurol 2012; 72: 335–343. 23034909 10.1002/ana.23635

[bib110] Cavanna A. The precuneus and consciousness. CNS Spectr 2007; 12: 545–552. 17603406 10.1017/s1092852900021295

[bib111] Nagahama Y, Okada T, Katsumi Y, Hayashi T, Yamauchi H, Sawamoto N et al. Transient neural activity in the medial superior frontal gyrus and precuneus time locked with attention shift between object features. Neuroimage 1999; 10: 193–199. 10417251 10.1006/nimg.1999.0451

[bib112] Simon O, Mangin J, Cohen L, Le Bihan D, Dehaene S. Topographical layout of hand, eye, calculation, and language-related areas in the human parietal lobe. Neuron 2002; 33: 475–487. 11832233 10.1016/s0896-6273(02)00575-5

[bib113] Berger B, Trottier S, Verney C, Gaspar P, Alvarez C. Regional and laminar distribution of the dopamine and serotonin innervation in the macaque cerebral cortex: a radioautographic study. J Comp Neurol 1988; 273: 99–119. 3209731 10.1002/cne.902730109

[bib114] Herrera-Marschitz M, Goiny M, Utsumi H, Ungerstedt U. Mesencephalic dopamine innervation of the frontoparietal (sensorimotor) cortex of the rat: a microdialysis study. Neurosci Lett 1989; 97: 266–270. 2654764 10.1016/0304-3940(89)90608-3

[bib115] Hillion J, Canals M, Torvinen M, Casado V, Scott R, Terasmaa A et al. Coaggregation, cointernalization, and codesensitization of adenosine A2A receptors and dopamine D2 receptors. J Biol Chem 2002; 277: 18091–18097. 11872740 10.1074/jbc.M107731200

[bib116] Borroto-Escuela D, Romero-Fernandez W, Tarakanov A, Ciruela F, Agnati L, Fuxe K. On the existence of a possible A2A-D2-β-Arrestin2 complex: A2A agonist modulation of D2 agonist-induced β-arrestin2 recruitment. J Mol Biol 2011; 406: 687–699. 21256133 10.1016/j.jmb.2011.01.022

[bib117] Volkow N, Wang G, Logan J, Alexoff D, Fowler J, Thanos P et al. Caffeine increases striatal dopamine D2/D3 receptor availability in the human brain. Transl Psychiatry 2015; 5: e549. 25871974 10.1038/tp.2015.46 PMC4462609

[bib118] Zhang S, Hu S, Chao H, Li C. Resting-state functional connectivity of the locus coeruleus in humans: in comparison with the ventral tegmental area/substantia nigra pars compacta and the effects of age. Cereb Cortex 2015; doi: 10.1093/cercor/bhv172; e-pub ahead of print. 10.1093/cercor/bhv172 PMC4961017 26223261

[bib119] Hou R, Freeman C, Langley R, Szabadi E, Bradshaw C. Does modafinil activate the locus coeruleus in man? Comparison of modafinil and clonidine on arousal and autonomic functions in human volunteers. Psychopharmacology (Berl) 2005; 181: 537–549. 15983798 10.1007/s00213-005-0013-8

